# Supplementary material for: Revealing the Increased Stress Response Behavior through Transcriptomic Analysis of Adult Zebrafish Brain after Chronic Low to Moderate Dose Rates of Ionizing Radiation
Source: Cancers (Basel). 2022 Aug 4;14(15):3793. doi: 10.3390/cancers14153793 (PMC9367516; doi:10.3390/cancers14153793)
Supplement: Supplementary file 1 [file cancers-14-03793-s001.zip › Table S2.pdf]

Table S2. Go terms associated to neurohormones or neurotransmitters for the volcano-plot of differentially expressed genes (DEG).

Neurohormones/ neurotransmitters	Go terms
Acetylcholine	GO:0008292, GO:0022848, GO:0015464, GO:0042166, GO:0005892, GO:0071340, GO:0016907, GO:0007197, GO:0005277, GO:0007213, GO:0033130, GO:1904395, GO:0003990, GO:0006581, GO:0030548, GO:0030550
Angiotensin	GO:0031701, GO:0038166, GO:0003081, GO:0002003, GO:0004945, GO:0001596, GO:0086097
Arginin-vasopressin	GO:0005000, GO:0001992
Corticotropin	GO:0004978, GO:0071376, GO:0043404, GO:0051424, GO:0051460, GO:1900011, GO:0051431, GO:0051429
Cortisol	GO:0051414, GO:0034650, GO:0034651
Dopamine	GO:0004968, GO:0097211, GO:0005183, GO:0031530
Epinephrin	GO:0001994, GO:0042421, GO:0032812, GO:0048242, GO:0071871, GO:0042415, GO:0005334, GO:0015874, GO:0051620
GABA	GO:0097154, GO:0004890, GO:0022851, GO:0051932, GO:1902711, GO:0032228, GO:0004965, GO:0050811
Glucocorticoid	GO:0051384, GO:0004883, GO:0042921, GO:0043402, GO:0006704, GO:0035259, GO:0031943, GO:0071385
Glutamate	GO:0004970, GO:0008066, GO:0035235, GO:0035249, GO:0004971, GO:0032281, GO:0098978, GO:0004972, GO:0017146, GO:0008328, GO:0015277, GO:0032983, GO:0007216, GO:0051966, GO:0035256, GO:0001641, GO:0007196, GO:0051967, GO:2000312, GO:0035255, GO:0051968, GO:0098990, GO:1905962
Gonadotropin	GO:0004968, GO:0097211, GO:0005183, GO:0031530
Hypocretin	GO:0031771, GO:0031772, GO:0042324
Melatonin	GO:0008502, GO:0030186, GO:0030187
Norepinephrine	GO:0001994, GO:0042421, GO:0042415, GO:0005334, GO:0015874, GO:0051620
Oxytocin	GO:0004990
Serotonin	GO:0004993, GO:0051378, GO:0007198, GO:0098664, GO:0005335, GO:0051610, GO:0006837, GO:0031821, GO:0042427, GO:0042428, GO:0007210
Thyrotropin	GO:0031531, GO:0004997, GO:0008437
